# Two genome sequences of the same bacterial strain, *Gluconacetobacter diazotrophicus* PAl 5, suggest a new standard in genome sequence submission

**DOI:** 10.4056/sigs.972221

**Published:** 2010-06-15

**Authors:** Adriana Giongo, Heather L. Tyler, Ursula N. Zipperer, Eric W. Triplett

**Affiliations:** Department of Microbiology and Cell Science, Institute of Food and Agricultural Sciences, University of Florida, PO Box 110700, Gainesville, FL 32611-0700 USA

**Keywords:** Optical Mapping, *Gluconacetobacter*, chromosomal rearrangements

## Abstract

*Gluconacetobacter diazotrophicus* PAl 5 is of agricultural significance due to its ability to provide fixed nitrogen to plants. Consequently, its genome sequence has been eagerly anticipated to enhance understanding of endophytic nitrogen fixation. Two groups have sequenced the PAl 5 genome from the same source (ATCC 49037), though the resulting sequences contain a surprisingly high number of differences. Therefore, an optical map of PAl 5 was constructed in order to determine which genome assembly more closely resembles the chromosomal DNA by aligning each sequence against a physical map of the genome. While one sequence aligned very well, over 98% of the second sequence contained numerous rearrangements. The many differences observed between these two genome sequences could be owing to either assembly errors or rapid evolutionary divergence. The extent of the differences derived from sequence assembly errors could be assessed if the raw sequencing reads were provided by both genome centers at the time of genome sequence submission. Hence, a new genome sequence standard is proposed whereby the investigator supplies the raw reads along with the closed sequence so that the community can make more accurate judgments on whether differences observed in a single stain may be of biological origin or are simply caused by differences in genome assembly procedures.

## Introduction

*Gluconacetobacter diazotrophicus* PAl 5 is a bacterial endophyte of sugarcane, originally isolated in Brazil [1] that provides fixed nitrogen to its plant host, in addition to increasing plant growth by mechanisms independent of nitrogen fixation [2,3]. The ability of *G. diazotrophicus* to increase growth and reduce plant dependence on nitrogen fertilization also makes it important to increasing the efficiency of biofuel production from sugarcane [4]. Since it was first isolated, additional strains of *G. diazotrophicus* have been isolated in several other countries and plant hosts [5-10]. As a result, there has been great interest in sequencing the genome of *G. diazotrophicus* to guide further research on this bacterium and to better understand endophytic nitrogen fixation by comparative genomics with other sequenced nitrogen fixing endophytic bacteria.

Genome sequences of *G. diazotrophicus* PAl 5 were recently completed by two groups, RioGene in Brazil, funded by FAPRJ, and the US DOE Joint Genome Institute (JGI) in California, USA. The RioGene sequence has been published [11].

Although both groups reported the genome sequence of the same strain, the two genome sequences vary between each other in gene arrangement and plasmid content, suggesting either that the original templates for genome sequencing were different strains of that sequencing and/or assembly errors exist in one or both of the genome sequences. Here optical mapping was used to elucidate which genome assembly is more closely related to the physical genome of PAl 5.

Optical mapping creates a physical restriction map of a genome assembled from DNA molecules immobilized on a glass slide prior to digestion with a selected restriction enzyme, maintaining the original order of restriction fragments. After digestion, DNA is stained and visualized by fluorescent microscopy, and the resulting digitized images are analyzed in an assembly program to construct an optical restriction map of the genome of interest [12,13]. These optical maps can be compared to *in silico* digests of DNA sequences and have been employed in many sequencing studies, serving as scaffolds for contigs alignment, as well as an independent means of identifying errors (inversions, insertions, deletions, translocations, etc.) in previously assembled sequences [14-20]. Therefore, optical mapping was deemed to be an ideal tool to elucidate which PAl 5 genome sequence most closely matched the physical DNA of the strain (ATCC 49037).

## Materials and Methods

### Bacterial strain

The bacterial strain used in this study is *Gluconacetobacter diazotrophicus* PAl 5 obtained from the American Type Culture Collection (ATCC 49037). *G. diazotrophicus* PAl 5 was cultured on yeast mannitol (YM) agar and broth at 30^o^C.

### Preparation of cells for optical mapping

*G. diazotrophicus* PAl 5 was grown in a 5 mL YM broth until the cells reached a density of 10^9^ CFU/mL. The culture was dispensed into five 1.5 mL microcentrifuge tubes in 1 mL aliquots. Tubes were then centrifuged at 6,000 rpm for 10 minutes to pellet the cells. Tubes with cell pellets were shipped on dry ice to OpGen Technologies, Inc. (Madison, Wisconsin) for optical mapping.

### Optical mapping and analysis

A BglII optical map of *G. diazotrophicus* PAl 5 was constructed by OpGen Technologies, Inc. (Madison, Wisconsin, USA). *In silico* BglII restriction maps of the two complete *G. diazotrophicus* PAl 5 genomic sequences on GenBank (GenBank # CP001189 and AM889285) were constructed from each sequence’s GenBank file and compared to the BglII optical map of PAl 5 using MapViewer version 2.1.1 (OpGen Technologies, Inc.). Plasmid sequences associated with each genome assembly did not align to the optical map and were therefore not included in the analysis.

### Comparison of annotation

The annotations of the two genomic assemblies were determined using RAST ver. 2.0 [21]. Genome and plasmid sequences for RioGene (GenBank# AM889285, AM889286, and AM889287) and JGI (GenBank# CP001189 and CP001190) were concatenated into single FASTA files prior to RAST analysis. Annotations determined by RAST were compared using the SEED viewer (ver. 2.0) (22) based on percent identity between coding sequences (CDS) and the functional roles assigned to annotated genes.

## Results

### Optical map of G. diazotrophicus PAl 5

A BglII optical map of *G. diazotrophicus* PAl 5 (ATCC 49037) was constructed in order to determine which genome assembly was the most accurate representation of the original strain. The optical map was 3,845,512 bp in length and composed of 424 restriction fragments, with an average fragment size of 9,070 bp (Table 1). In comparison, the *in silico* map of the JGI sequence was 3,887,492 bp in length, while the RioGene map was 3,944,163 bp (Table 1). The average fragment length of both *in silico* maps is over 1,000 bp shorter than the average fragment length of the optical map (Table 1). These differences between the optical and *in silico* maps are likely due to the fact that restriction fragments shorter than 500 bp are not detected by optical mapping owing to such short fragments being washed off the optical slide [22].

**Table 1:** Optical and *in silico* BglII restriction maps for *G. diazotrophicus* PAl 5

	**Optical Map**	***In silico* BgIII restriction map**	
		**JGI**	**RioGene**
Map Length (bp)	3,845,512	3,887,492	3,944,163
Number of Fragments	424	486	503
Average fragment length (bp)	9,070	7,999	7,841
Maximum fragment length (bp)	52,064	51,728	50,690
Minimum fragment length (bp)	562	24	28

### Identification of sequence rearrangements using optical mapping

**Figure 1:**
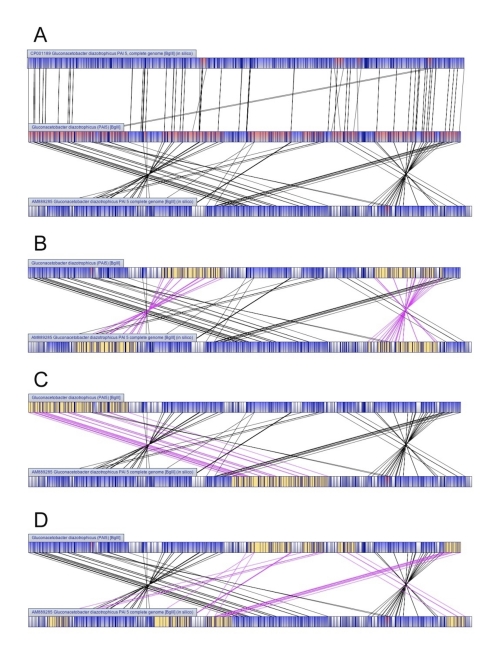
- Alignment of *G. diazotrophicus* PAl 5 optical map with in silico maps of genome sequences **A**: The BglII optical map of *G. diazotrophicus* PAl 5 aligned against *in silico* optical maps calculated from the genome sequence proposed by RioGene (AM889285) and JGI (CP001189). **B-D**: Misassemblies in PAl 5 RioGene sequence when aligned against the optical map. **B**: Two large inversions in RioGene sequence compared to the optical map. **C**: Large translocation in RioGene sequence. **D**: Five translocations in RioGene sequence. Dark blue represents cut sites, light blue represents aligned regions, red represents regions aligning to both sequences, and white represents unaligned regions. Alignment lines for inversions and translocations highlighted in pink. Inverted and translocated regions highlighted in yellow.

Once the BglII optical map of PAl 5 was aligned to *in silico* BglII restriction maps generated from the two separate genome sequences, it was readily apparent that the sequence from RioGene contained numerous chromosomal rearrangements relative to the physical map (Figure 1a). Comparison of the optical map to the RioGene *in silico* map revealed the presence of two large inverted regions (Figure 1b). These inversions were 555.9 and 564.3 kb in length, together spanning close to 28% of the genome sequence (Table 2). In addition, numerous translocations were identified in the RioGene sequence. One large translocation spanning 865.8 kb of the genome (Figure 1c) and 5 smaller translocations ranging in size from 69.8 to 330.8 kb (Figure 1d) were identified (Table 2). From these determinations, it appears that 74% of the PAl 5 genome sequence proposed by RioGene is rearranged compared to the physical map of the PAl 5 genome. In contrast, the *in silico* map of the JGI PAl 5 sequence showed a higher alignment to the optical map (Figure 1a). Only three small inversions and one small translocation were detected, covering only 5.6% of the genomic sequence.

**Table 2:** Rearrangement positions in *G. diazotrophicus* PAl 5 genome sequence from RioGene

**Rearrangement type**	**Location in genome (bp)**		**Length (bp)**
	**Start position**	**Stop position**	
Inversion	391,267	955,614	564,347
	3,078,324	3,634,241	555,917
Translocation	149,268	358,682	209,414
	1,115,930	1,446,765	330,835
	1,581,823	1,651,595	69,772
	1,627,253	1,796,352	169,099
	1,796,352	2,662,168	865,816
	3,706,896	3,878,171	171,275

Additionally, several regions of the RioGene BglII *in silico* map did not align to the optical map (Figure 1a). Together, these regions totaled 1,053,347 bp, or 26.7% of the genome sequence (Table 3). In comparison, the regions of the JGI *in silico* map that did not align to the optical map were composed largely of single restriction fragments, most of which were 500 bp or less (Table 3), a length that is below the detection threshold of optical mapping technology. Thus, it appears that much of the PAl 5 genome sequence from RioGene was either rearranged or did not align to the optical map of PAl 5.

**Table 3:** Regions of *in silico* maps not aligned to the *G. diazotrophicus* PAl 5 optical map

**Length of the fragments (bp)**	**JGI**	**RioGene**
Total length of unaligned regions	27,540	1,053,347
Average unaligned fragment length	574	5,885
Maximum unaligned fragment length	1,341	32,719
Minimum unaligned fragment length	24	28

### Differences in annotation between genome sequences

Given the high level of chromosomal rearrangements and non-aligned regions between genomic sequences reported from the same strain, the annotations of both PAl 5 sequences were determined using the Rapid Annotation using Subsystem Technology (RAST) web based annotation service [21] to ascertain what effect these rearrangements have on gene calling. With a total of 8 rearrangements in the RioGene sequence, there are up to 16 locations were coding sequences (CDS) could have been disrupted. Interestingly, 168 and 187 of the CDS identified in the RioGene and JGI genomes, respectively, were unique, sharing zero percent identity with CDS in the other genome (Table 4). In total, 247 of the CDS in the RioGene sequence shared less than 50% identity with CDS in the JGI sequence (Table 4). This number of differences between the two genome sequences was over 10 times greater than expected from the observed inversions and translocations in the RioGene sequence. Since both genomic sequences are reportedly from the same ATCC strain, a similar complement of genes was expected. However, only 90% of the CDS predicted in each genome shared greater than 90% identity (Table 4).

**Table 4:** Comparison of coding sequences between *G. diazotrophicus* PAl 5 genome sequences based on percent identity

**Percent identity to** **comparison genome**	**JGI**		**RioGene**	
	**Number of CDS**	**Percent of total CDS**	**Number of CDS**	**Percent of total CDS**
100	2024	56.7	2069	56.0
³ 99	2812	79.2	2876	77.8
³ 90	3190	89.8	3313	89.6
> 75	3267	92.0	3402	92.0
³ 50	3326	93.7	3449	93.3
< 50	225	6.3	247	6.7
0	187	5.3	168	4.5

Fewer differences were observed between the genome sequences when the functional roles of genes were examined. In total, 13 and 21 of the functional roles identified were unique to the RioGene and JGI sequences, respectively (Table 5). Given the number of inversions and translocations in the RioGene sequence, the annotation was also checked for transposases that could potentially contribute to chromosomal rearrangements. The RioGene PAl 5 sequence was found to possess 110 transposase genes, while the JGI sequence only contained 59 transposase genes. A large number of these were putative transposases, though several IS3, IS4, and IS5 family proteins were also identified (Table 6). Only three translocated regions had transposases within 10 kb from either end, indicating other factors may have contributed to the rearrangements observed between the sequences.

**Table 5:** Unique functional roles between *G. diazotrophicus* PAl 5 genome sequences

**Roles Unique to JGI**	**Roles Unique to RioGene**
Ribose ABC transport system, periplasmic ribose-binding protein RbsB (TC 3.A.1.2.1)	Sorbitol dehydrogenase (EC 1.1.1.14)
	
	
	
	
	
D-alanine--D-alanine ligase (EC 6.3.2.4)	Transketolase, C-terminal section (EC 2.2.1.1)
	
	
	
	
	
UDP-N-acetylenolpyruvoylglucosamine reductase (EC 1.1.1.158)	Transketolase, N-terminal section (EC 2.2.1.1)
	
	
	
	
	
Organic hydroperoxide resistance protein	COG0028: Thiamine pyrophosphate- requiring enzymes
	
	
	
	
	
Organic hydroperoxide resistance transcriptional regulator	D-galactonate regulator, IclR family
	
	
	
	
	
	
Molybdenum cofactor biosynthesis protein B	Epi-inositol hydrolase (EC 3.7.1.-)
	
	
	
	
	
Flagellar biosynthesis protein fliL	Chromosome partition protein smc
	
	
	
	
	
Flagellar hook-associated protein flgL	dTDP-rhamnosyl transferase RfbF (EC 2.-.-.-)
	
	
	
	
	
Deoxyuridine 5’-triphosphate nucleotidohydrolase (EC 3.6.1.23)	Protein of unknown function DUF374
	
	
	
	
	
Aminopeptidase S (Leu, Val, Phe, Tyr preference) (EC 3.4.11.-)	Nicotinate-nucleotide adenylyltransferase (EC 2.7.7.18)
	
	
	
	
	
Leucyl/phenylalanyl-tRNA—protein transferase (EC 2.3.2.6)	DNA repair exonuclease family protein YhaO
	
	
	
	
	
Cysteinyl-tRNA synthetase (EC 6.1.1.16)	ATP-dependent DNA helicase UvrD/PcrA, proteobacterial paralog
	
	
	
	
	
tRNA:Cm32/Um32 methyltransferase	Outer membrane lipoprotein carrier protein LolA
	
	
	
	
	
	DNA-binding response regulator KdpE
	
	
	
	
	
	Osmosensitive K+ channel histidine kinase KdpD (EC 2.7.3.-)
	
	
	
	
	
	Potassium-transporting ATPase A chain (EC 3.6.3.12) (TC 3.A.3.7.1)
	
	
	
	
	
	Potassium-transporting ATPase B chain (EC 3.6.3.12) (TC 3.A.3.7.1)
	
	
	
	
	
	Beta-hexosaminidase (EC 3.2.1.52)
	
	
	
	
	
	Potassium-transporting ATPase C chain (EC 3.6.3.12) (TC 3.A.3.7.1)
	
	
	
	
	
	Protein-export membrane protein secD (TC 3.A.5.1.1)
	
	
	
	
	
	H^+^/Cl^-^ exchange transporter ClcA

**Table 6:** Transposases in *G. diazotrophicus* PAl 5 genome sequences

	**JGI**	**RioGene**
Total transposase genes	59	110
Transposase	6	19
Transposase (class II)	1	2
Transposase (class III)	1	0
Transposase (class IV)	1	0
Putative transposase	27	64
Transposase IS3 family protein	2	4
Transposase IS3/IS911 family protein	1	0
Transposase IS4 family protein	6	4
Transposase IS5 family protein	4	7
Transposase IS256	1	0
Transposase IS630	0	1
Isrso16-transposase OrfA protein	1	0
Transposase and inactivated derivative	2	1
Transposase mutator type	5	6
Probable insertion sequence transposase protein	1	0
TRm2011-2a transposase	0	2

## Discussion

The construction of two different genome sequences from the same bacterium, *G. diazotrophicus* PAl 5 (ATCC 49037), demonstrated the need to confirm the sequence and assembly of these genomes through an independent method. In the current study, optical restriction mapping was used to distinguish between the discordant genomic assemblies, since this technique maintains the order of restriction fragments in the mapping process.

When comparing, the two PAl 5 genome sequences to an optical map, the resulting analysis led to the determination that the sequence reported by JGI is a more accurate representation of the PAl 5 strain (ATCC 49037) while the sequence reported by RioGene contained numerous rearrangements. The size and number of chromosomal rearrangements identified in the RioGene sequence of *G. diazotrophicus* PAl 5 was high, with nearly the entire sequence composed of regions that were inverted, translocated, or not aligned to the PAl 5 optical map. In contrast, only a few small inversions were detected in the JGI PAl 5 sequence. In addition, annotation of the two genome sequences found that approximately 5% of the CDS in each genome sequence were unique. This is a surprisingly high amount considering the two genomes are reported to be from the same strain and much greater than would be expected from the observed sequence rearrangements.

There are a few possibilities for the differences between these two PAl 5 genome sequences. One explanation is natural divergence due to rapid evolution that could occur during culturing. This explanation was also suggested by the RioGene sequencing group [11]. However, the extremely high level of differences between the two sequences indicates other factors may have also contributed. For example, in the case of *E. coli*, comparison of the sequenced K-12 strain to the optically mapped H10407 strain revealed no major structural differences [23]. In the case of *M. avium* subspecies *paratuberculosis*, only one inversion between the sequenced strain, K-10, and the optically mapped strain, ATCC 19698, was detected, and that inversion was subsequently determined to be an assembly error rather than a true chromosomal rearrangement [17].

Another explanation for the differences between the RioGene and JGI sequences is the different approaches taken by the two groups in genome assembly. To test this possibility, the raw reads from both projects are required. The 46,603 sequence traces from the JGI sequencing effort of this strain are publicly available while the traces from the RioGene project are not. The quality scores of the bases are not available from either project. While many studies have reported using optical maps to aid in genome assembly and identification of assembly errors prior to completion, fewer have reported using this technique to identify errors in previously completed genomes. After successfully using optical mapping to aid in assembling the genome of *Xenorhabdus nematophila*, Latreille *et al.* [14] used the same technique on a previously sequenced relative, *X. bovienii*, identifying a large inversion in the genome assembly that had previously been considered finished. In addition, optical mapping has also been used to verify assemblies between strains of the same species. In the case of *Mycobacterium avium* subspecies *paratuberculosis*, an optical map of the ATCC type strain was used to reveal the presence of an inversion in the genome of the sequenced strain, which was determined to be due to an assembly error rather than genomic variation between strains [17]. These two instances illustrate how even complete, published genome sequences may contain significant assembly errors, indicating that caution should be taken when looking at assemblies where optical mapping was not utilized.

If the breakpoints of assembly errors occur within a coding region, such errors could alter the annotation of the genome. For example, when the inversion in the sequence of *M. avium* subspecies *paratuberculosis* K-10 was corrected, two new genes were identified [17]. Therefore, the annotation of the PAl 5 sequences from both RioGene and JGI were determined and compared using the RAST on-line annotation pipeline [21]. Six percent of the CDS from each genome shared less than 50% identity when compared against each other and approximately 5% shared zero percent identity. Again, this number of differences at the sequence and gene level was surprising considering the genomes are reported from the same strain, even given chromosomal rearrangements. Annotation of both genomes also revealed that the RioGene sequence possessed almost twice as many transposases as the JGI sequence. The strikingly high number of transposases in the RioGene sequence in relation to the JGI sequence suggests the possibility that some of the sequence rearrangements seen may be the result of transposition. Alternatively, since 16 of the transposases originated from IS sequences, which are flanked by inverted repeats [24], there is also the possibility that these repeated regions caused errors in assembly.

The observations made here confirm the utility of optical mapping in determining proper assembly of genomic sequences and identifying potential chromosomal rearrangements. It also highlights the need to provide raw reads and quality scores when submitting genomes to allow for independent confirmation of assembly. As technology advances, data from instances where contradictory sequences are observed could be reanalyzed in order to clarify results.

The rearrangements in the genome sequence of *G. diazotrophicus* PAl 5 may not have been identified had JGI not released a conflicting genome sequence of the same strain that prompted further investigation. As sequencing the genome of a single bacterial strain is not usually performed separately by different groups, the possibility remains that other previously released genomes could contain similar differences compared to other bacterial isolates under the same ATCC strain designation. Such rearrangements in genome sequences of the same strain could confound future work using comparative genomics to look for variations between closely related organisms. In such cases, the best tool to distinguish actual variations between organisms will be optical mapping. Consequently, the submission of raw sequencing reads with quality scores is proposed as a new genome sequencing standard when submitting completed genomes to GenBank or other repository.
